# Microbiome and Colorectal Cancer Management

**DOI:** 10.7759/cureus.30720

**Published:** 2022-10-26

**Authors:** Mahmoud Alrahawy, Saryia Javed, Haitham Atif, Kareem Elsanhoury, Kamel Mekhaeil, George Eskandar

**Affiliations:** 1 General Surgery, Menoufia University, Menoufia, EGY; 2 General Surgery, Queen Elizabeth University Hospital, Glasgow, GBR; 3 Vascular Surgery, James Cook University Hospital, Middlesbrough, GBR; 4 Surgery, James Cook University Hospital, Middlesbrough, GBR

**Keywords:** colorectal cancer, target therapy, diagnosis, biomarker, crc, cancer, colorectal, microbiome

## Abstract

Globally, colorectal cancer (CRC) is one of the most typical lethal cancers. One of the main factors for better outcomes in CRC management is the early detection of the disease. As an integral component of human metabolism and homeostasis, gut microbiome has recently been a subject of extensive research for its role in the pathogenesis, diagnosis, and treatment of CRC.

Microbial dysbiosis (the decrease in beneficial gut flora and the increase of detrimental populations) leads to chronic inflammation and genetic alteration in the host cells, triggering and promoting CRC carcinogenesis. Identifying these microbial changes in depth would potentially isolate the pathogenic microbiota species and establish biomarker models for early detection of CRC. On the other hand, modifying these microbial changes would help formulate preventative and therapeutic strategies for CRC, developing a more precise CRC management plan according to each patient's microbial print. This essay explains gut microbiome composition, microbial changes (dysbiosis) in CRC carcinogenesis, the probability of creating microbiome-based CRC biomarkers, and potential microbiome-targeted treatment options.

## Introduction and background

Colorectal cancer (CRC) is the fourth most common cancer in the UK and accounts for 12% of all new cancer cases [1]. Considered a "silent disease", early diagnosis of CRC is a chief factor that influences survival; therefore, establishing reliable non-invasive CRC biomarkers is required [2].

Currently available CRC screening options include faecal occult blood tests (FOBTs), faecal immunochemical tests (FITs), and subsequent colonoscopy if FOBT or FIT are positive [3]. FOBT's sensitivity is still limited because CRCs do not always bleed or only bleed intermittently [4]. While colonoscopy represents the standard gold method for diagnosing CRC, it is challenging to use it as a screening test due to its high cost and invasiveness. Thus, there is a need to find other feasible CRC screening methods [5].

The intestinal microbiome, gut flora, had primarily been hidden in the blind spot of the medical research community until the last 10 years [6]. There is a rapid proliferation of interest in studying this "forgotten organ" and correlating its function to several human pathological changes, such as cancers, especially after the advances in DNA/RNA sequencing [7]. Current evidence support that specific intestinal microbes drive CRC development and progression, yet their pathogenic mechanisms are still unclear. Microbial analysis can also identify some faecal microbial markers of CRC that could help in the early diagnosis [8].

This study aims to explore some information about the microbial community and how microbiota changes could trigger CRC pathogenesis, the different techniques for microbial analysis, and the potential use of microbiome as a biomarker to detect CRC and as an adjunct component of its treatment.

## Review

Gut microbiota composition

Symbiotic Microbial Composition

Gut microbiota includes the microorganisms, such as bacteria, viruses, archaea, and eukaryotic organisms, that inhabit the human gastrointestinal tract and manipulates several physiological functions [9]. Meanwhile, microbiome refers to the gut microbiota's collective genes, genomes, and metabolic products in the host environment [10]. Holobiont refers to the biological entity involving a host and its inherited symbiont microbiota, while hologenome describes the collective genomes of the host genome and associated microbial genomes [11]. A healthy gut microbiota composition (symbiosis or eubiosis) is essential for maintaining normal gut nutrition, metabolism, cell proliferation, immune system development, and protection against pathogenesis [12].

The microbial structure is affected by various factors, such as dietary carcinogens, smoking, alcohol, and other environmental factors. Chief among factors affecting gut microbiome composition is the modern western lifestyle (associated with increased fast food and stress levels) that leads to reduced beneficial bacteria and enriched pathogenic species [13]. The high calorific content of the western diet (high fat and carbohydrates) causes microbial structure changes (dysbiosis) and increases the risk of developing obesity [14] and carcinogenesis [15]. On the other hand, fasting can increase the diversity of bacteria (symbiosis) in your gut, which is essential for your immune and overall health [16]. Cignarella et al. have reported that intermittent fasting, ideally 16 hours of fasting and 8 hours of diet, resulted in increased enrichment of the* Lactobacillaceae *bacteria families (probiotic) that have beneficial effects on health, including the exclusion of pathogens, immunomodulation, and the production of a healthy bacterial molecule [17,18]. Intermittent fasting also has potent immunomodulatory effects that are at least partially mediated by the gut microbiome [19].

Microbial Dysbiosis

Microbial dysbiosis refers to pathological alterations of gut microbiota compositions, resulting in several disease states [15]. Dysbiosis has been associated with a wide range of diseases, including type 1 diabetes mellitus, inflammatory bowel disease (IBD), allergic disorders, metabolic syndrome, non-alcoholic fatty liver disease, obesity, and CRC in both human and animal models [20-22]. One of the necessary consequences of gut microbial dysregulation is cancer development due to an increased percentage of harmful bacterial microbiota that produces pro-carcinogenic substances and destroys the gut barrier [23]. Microbiota-induced inflammation and genotoxicity eventually induce carcinogenesis and the development of CRC [24].

Microbiome and CRC interplay

Correlation between gut microbial changes and CRC formation has been established in several studies recently [25-28] because of up-to-date gene sequencing techniques [26]. Microbiota within tumour tissue has a specific bacterial composition compared to normal healthy areas. Bacterial species such as *Bacteroides fragilis*, *Escherichia coli*, and *Fusobacterium nucleatum* are more abundant in the CRC microenvironment [29-31], and this potentially could drive using microbiota as CRC biomarkers [32].

Mechanism of CRC Development by Microbial Dysbiosis

Many recent studies have highlighted the link between CRC and gut microbiome alteration [29], with several hypotheses as to the causal role of microbes in CRC development [33]. Yet, the exact pathogenic mechanisms are still unclear [28]. Gut microbiota could influence colorectal carcinogenesis through various mechanisms that are described below.

**Figure 1:**
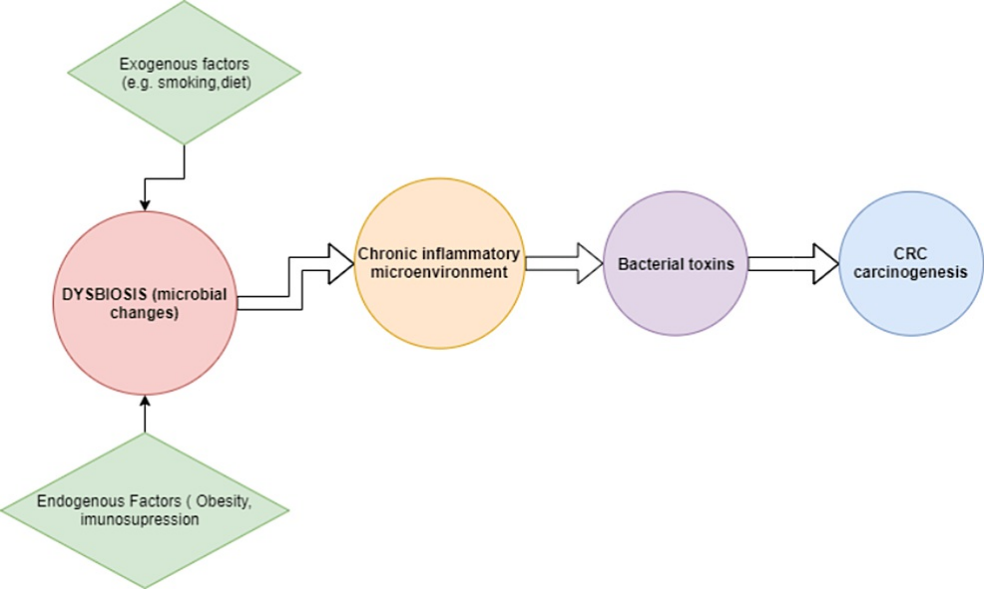
Dysbiosis-induced CRC development CRC: colorectal cancer.

Microbial-induced inflammation: Some evidence suggests that gut microbiota induce chronic inflammation, which might trigger carcinogenesis [34]. Zhang et al. reported that CRC-associated microbial changes and subsequent gradual inflammation might gradually form a microenvironment that triggers CRC [35]. *F. nucleatum*, a common oral bacterium, has also been associated with CRC progression, metastasis, and chemoresistance [36]. As a pro-carcinogenic bacterium, *F. nucleatum* codes a virulence factor, FadA, that can activate the β-catenin pathway, which drives the initiation and progression of CRC. *E. coli* has also presented abundance in CRC with a significant role in promoting neoplasia [37]. Figure 1 explains how the microbiome could produce inflammatory changes that initiate CRC development.

Nevertheless, there is not enough evidence to say that inflammation or the presence of bacteria or bacterial metabolites alone is enough to promote tumour growth [38]. However, it is still an active area of inquiry to determine which microbial species are most responsible for carcinogenesis.

Driver-passenger model: Two classes of the microbial community are involved in CRC pathogenesis, namely, the "driver" community and the "passenger" community [39]. The "driver" community (pro-carcinogenic indigenous bacteria) cause microenvironmental alterations that potentially initiate carcinogenesis by producing carcinogenic toxins that damage DNA in colonic epithelial cells [17]. *Enterococcus faecalis* represents an example of a driver microbiome as it releases extracellular superoxide that causes intracellular DNA carcinogenic alterations within the colonic mucosa [17]. Subsequently, the "passenger" community (opportunistic colonising bacteria) outcompetes the driver microbes and augments tumour progression and growth. The commonest passenger bacteria to colonise in CRC tissue is *Fusobacterium* spp. [39].

Host-microbial genetic interaction: Traditional theories of cancer aetiology focus on the mechanism of altering mammalian genetics by external risk factors such as smoking [9]. However, with advanced computerised diagnostics, internal host-microbial genetic interactions have been of study interest [40]. There is evidence that host-to-microorganism interactions activate procarcinogenic signalling pathways that trigger molecular alterations (genomic and epigenetic changes), stimulation of adenoma-carcinoma sequence, and then CRC development and progression [41].

Recently, host-microbe interactions in CRC tumours have been studied to prove an association between specific tumour mutations and distinct microbial, metabolic, and interaction profiles [42]. Burns et al. also found statistically significant associations between loss-of-function mutations in tumour genes and shifts in the abundances of specific bacterial taxa, suggesting a potential functional genetic interaction between bacteria and tumour profiles [28].

These mechanistic components have the potential to be modulated for therapeutic or prophylactic purposes in the context of CRC. Nevertheless, these studies only show correlations and cannot directly cause effects. Thus, it is unclear whether the microbiome is altered before or after the appearance of specific mutations.

Microbial analysis techniques

Most human microbial bacteria are uncultivable, so microbiome studies on CRC patients have relied on molecular-based methods [39]. Targeted genotyping (e.g. 16S ribosomal RNA (rRNA) based) and metagenomics are the most widely utilised methods for microbial analysis.

Gene Amplicon Sequencing

Over the past few decades, gene amplicon sequencing has been the primary technique for studying microbial-specific marker/target genes that function in evolutionary transitions and changes [43]. 16S rRNA sequencing is the gold standard targeted gene sequencing approach for identifying microbial community composition and assessing genetic diversity, especially for unculturable organisms [44]. Moreover, 16S rRNA gene sequencing analysis has assisted in correlating between alterations in microbial community (dysbiosis) functions and certain diseases, including Crohn's disease, ulcerative colitis, diabetes, and gastrointestinal cancers [45-47]. However, the 16S rRNA gene sequences approach does not target the whole genome realistically. It is limited in assessing molecular host-microbiota and microbiome-microbiome interactions that reflect the biological microbial community [48].

Metagenomics

Metagenomics comprehensively catalogues all microorganisms present (unculturable and culturable, known and unknown) in complex environmental samples [49]. The metagenomic analysis provides a functional analysis of microbial communities, such as polygenetic analysis and taxonomic classification. As a result, metagenomics outperforms 16S rRNA target gene sequencing in defining microbiota ecosystems, which gives a unimodal single-gene analysis [50].

Metagenomics is also reliable for studying the microbial community's genomic linkages between function and phylogeny (evolutionary history and relationships among or within groups of organisms) [50]. Metagenomics, complementing metatranscriptomic or metaproteomic techniques, could describe more expressed microbial activities [51].

Current studies focus mainly on the identification and profiling of microbial composition. Still, the microbial community is more complex and requires including the molecular interactions with the host and in-between microbiota to reflect the actual biological microenvironment [52].

Limitations of microbial analysis

Methodological Limitations

Current microbial analysis designs have some challenges. Amongst them is the absence of a gold-standard unified methodology of studying. The sample size is usually tiny and variable from one centre to another, which produces the most non-reliable and non-representative outcomes. Samples are mainly collected from faecal content without mucosal biopsies, which partially reflect the gut microbiome community [53]. Other challenges of getting an effective sample include handling (e.g. type (faecal or mucosal), collection, contamination, transportation, storage, and time to analysis), and nucleic acid analysis (e.g. methods for DNA extraction, selecting regions and depths of sequencing, varying polymerase chain reaction (PCR) primers for 16S microbial analysis, and variable methods for assignment of taxonomy) [54].

Gut microorganisms are considered difficult to culture [55]. Non-bacterial microbial components (i.e. virome, mycobiome, and protozoans) are less studied in current research due to a lack of facilities, despite having an established role in CRC stage progression [54]. Faecal samples are a non-invasive approach for screening tests; however, mucosal samples are more potent for identifying specific species of bacteria associated with CRC initiation and growth [56].

The lack of unified metadata and high-processing computer equipment, for high-volume data and statistical analysis, also affects the results' accuracy, reproducibility, and interpretability. Advanced computational tools for functional analysis that consider host-microbial molecular interactions are required to get more reliable results [57].

Exogenous and Endogenous Variability

Another challenge is that case-control studies are affected by the host (age, sex, and genotype) and environmental (e.g. diet and lifestyle) factors that produce biased results [58]. The existing database also focuses only on the western population that does not consider the heterogenicity of these variables. Therefore, it is crucial to include these factors, such as diet, lifestyle, and smoking, when examining microbiota's role in CRC and other diseases [59]. Therefore, some studies suggested a new strategy for microbial analysis in CRC by using tumour and normal tissue samples from the same CRC patient [60,61]. Burns et al. [62], for example, have found different microbial communities with specific functional pathways in CRC tissue compared to nearby healthy tissue. Therefore, it is essential to include these exogenous and endogenous differences with personalised study patterns to set reliable human microbial datasets (Table 1) [57].

**Table 1:** Challenges and required developments for better microbial outcomes in CRC CRC: colorectal cancer; PCR: polymerase chain reaction.

Methodology	Limitation	Development
Population sample	Heterogenicity (i.e. geography and lifestyle); most current studies include western populations [59].	Comprehensive and diverse studies for a better microbial database [57].
Study design	Case-control: Controls are affected by host and environmental factors (i.e. diet and genetics) [60].	Individualised approach (i.e. paired diseased-healthy tissue samples, diet, and metabolic analysis) for a personalised diagnosis and personalised therapy (i.e. pre/probiotics) [63].
Sample collection	Faecal samples (partially reflect gut microbiome) [54].	Tissue (mucosal) samples for a better understanding of environmental processes and biological interactions [64].
Microbial sample	Non-bacterial microbial components (i.e. virome, mycobiome, and protozoans). Non-bacterial microbial dataset (limited): gut virome dysbiosis (differ in CRC stages, patients - control) [54].	Taxonomy-based analysis: CRC-associated bacterial taxa + less covered taxonomic groups (i.e. fungi and viruses) [23]. Distinct sequencing techniques (i.e. regions, depth, and PCR) avoid heterogenicity bias [57].
Data analysis	Independent taxons analysis: Without considering ecological correlation -> decretive host-microbial interaction [65].	Functional-based analysis: Combined omics (i.e. metagenomic, metatranscriptomics, metaproteomics, and metabolomics) approaches of a mechanistic host-microbial interaction for direct causal effect [23].

Future Challenges

To get more specific bioinformatics about CRC-related microbiota, studies should involve the environmental functions and interactions within the microbial micro-environment along with the anatomical compositions [62]. In recent research, omics datasets (genomics, transcriptomics, proteomics, metabolomics, metagenomics, phenomics, etc.) have been included to describe microbial biological processes more accurately. Metatranscriptomics assess microbiota communities' taxonomic signature and function; meanwhile, metaproteomics analyses the microbiome-associated protein profiles to reflect the bodily functions under different environmental conditions [66]. Multi-omics data analysis requires highly advanced computational and technological resources with complex algorithms and software to process a high volume of data and correlate multiple variables and interactions [67]. These data would explain specific biological and environmental interactions that describe microbiomes' role in CRC development and create better therapeutic and biotechnological applications.

Clinical applications of microbiota in CRC

Microbial Biomarkers

Several biomarkers (genetic, blood, molecular, and imaging biomarkers ) are crucial in early detection, prognostication, and risk stratification in CRC [68]. Getting clinically reliable predictive biomarkers could improve the accuracy of predicting clinical outcomes such as survival, tumour recurrence, and metastasis. Personalised CRC treatment algorithms would be applied in clinical practice to get the best results for each individual's disease characteristics [69]. Studying microbial changes, therefore, could provide a diagnostic and predictive CRC biomarker soon [70].

Recent studies suggest that gut microbiome analysis of stool samples significantly differentiated between healthy individuals and patients with adenoma vs. carcinoma samples by identifying either the enrichment or depletion of specific bacterial populations within faecal samples [71]. Subsequently, the gut microbiome could serve as a screening tool for CRC detection [72]. By combining microbial biomarker tests with the FITs, sensitivity for CRC detection and accuracy of treatment outcomes prediction might increase [73].

The faecal metabolome is the metabolites, such as short-chain fatty acids, produced from microbial interactions to maintain the homeostasis of host metabolism [74]. Metabolomic analysis of faecal samples might also be potential clinical CRC biomarkers [36]. Xinhao et al. concluded that an increased abundance of specific gut microbiota (e.g. *B. fragilis*) was significantly associated with increased levels of particular metabolites (called metabolome), such as adrenic acid and decanoic acid, in CRC patients [75]. Faecal metabolomic analysis has produced some identified biomarkers for CRC diagnosis and therapeutic evaluation; however, more research studies are necessary to get more data on metabolomes [76].

Local volatile organic compound (VOC) is another potential CRC biomarker that reflects gut microbial compositions [77]. It is the gaseous molecules produced during bacterial fermentation in the gut and then emitted from urine and faeces that have shown specific signatures that reflect gut microbial compositions and functions [78]. With gas chromatography-mass spectrometry (GC-MS), faecal or urinary VOC analysis could also serve as a novel screening tool for CRC detection [79-81]. Vernia et al., in a multicentric study, recruited CRC patients screened with colonoscopy to analyse VOC in their breath. They reported that VOC models could detect patients with CRC with an area under the curve (AUC) of 0.84 with 95% sensitivity and 64% specificity [82]. VOC analysis, therefore, represents a promising non-invasive tool for CRC screening [82].

Gut Microbiome and CRC Precision Therapy

Modern microbial analytical techniques have improved our understanding of the mechanism of CRC formation linked to dysbiosis [83]. It is hypothesised that using antibiotics may also result in microbial dysbiosis and immune system issues, which speed up CRC progression [84]. Therefore, reversing these dysbiotic changes may aid in preventing and treating them [85]. Potential therapeutic strategies include dietary changes, pre/probiotics, faecal microbiota transplantation (FMT), and antibiotics [86].

**Figure 2:**
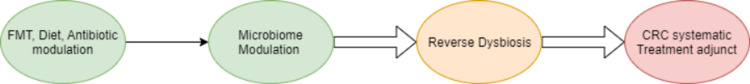
Microbiome modulation and CRC treatment FMT: faecal microbiota transplantation; CRC: colorectal cancer.

Some studies suggest that dietary modifications, including ingesting more fibre, may reduce the incidence of colon cancer and work in conjunction with traditional treatments [87-89]. Probiotics, live beneficial flora, may treat CRC by combating CRC-driver microbiota, increasing gut microbial diversity [90], improving immunity homoeostasis, reducing chronic inflammation, and decreasing carcinogenic metabolites [87]. Through gut microbiome reconstruction, FMT can improve bile acid metabolism and immunotherapy efficacy and subsequently serve as a natural remedy for CRC [91]. A healthy environment, good food, exercise, weight control, and avoiding or alleviating stress with relaxation techniques are also essential preventative strategies against developing CRC (Figure 2) [85]. Obesity, diabetes, irritable bowel syndrome, IBD, depression, and cardiovascular disease are also studied for microbiome-based therapy [92-97].

Future studies will need to improve ways for modulating the microbiome and provide major unsolved questions (e.g. CRC-microbiota causal link) that will be addressed in existing and future research [98]. Combining endogenous host variables (e.g. host genetics and the microbiome) with exogenous environmental factors (e.g. nutrition and smoking) can significantly impact the treatment response of CRC [9].

## Conclusions

A substantial body of research has established a strong relationship between microbial alterations (dysbiosis) and CRC carcinogenesis. However, the precise microbial-host interactions in CRC development remain elusive and influenced by various cofactors (exogenous and endogenous).

Some studies have expressed that microbiome alterations can be modified to treat CRC. However, more sophisticated molecular-based analysis and prospective interventional studies may yield more specific CRC microbial biomarkers and personalized therapeutic techniques for CRC management.
